# Using Systematic Observation and Polar Coordinates Analysis to Assess Gender-Based Differences in Park Use in Barcelona

**DOI:** 10.3389/fpsyg.2018.02299

**Published:** 2018-11-27

**Authors:** Félix Pérez-Tejera, Sergi Valera, M. Teresa Anguera

**Affiliations:** ^1^Department of Social Psychology and Quantitative Psychology, Faculty of Psychology, University of Barcelona, Barcelona, Spain; ^2^Social Environmental and Organizational Psychology Research Group (PsicoSAO), Barcelona, Spain; ^3^Institute of Neurosciences, University of Barcelona, Barcelona, Spain

**Keywords:** park use, public space, gender perspective, systematic observation, lag sequential analysis, polar coordinate analysis

## Abstract

This paper aims to assess gender differences in the usage of public open spaces (POS), as an everyday context. Forty POS in the city of Barcelona were studied over 3 months using systematic observation. To objectively measure park use, an observational instrument (EXOdES) was purposely designed combining a field format and several category systems. The instrument facilitated the record of configurations or co-occurrences of codes from different dimensions (i.g., time of day, age, race/ethnicity, activity setting, activity, and presence of vehicles), providing contextually rich data of more than 35,000 individuals and groups and the setting in which the activity occurs. Although a similar overall proportion of males and females were found using POS (55 vs. 45%), important differences by gender were found between people being alone (66 vs. 34%), and groups (53 vs. 47%). To identify regular patterns in the way that men and women use public parks, information on more than 18,000 groups of people was analyzed as a global data set. A multievent sequential analysis was performed considering gender composition as the given behaviors (i.e., groups of males, females, and gendered mixed). Thus, polar coordinates analysis was also performed, because it is a suitable reduction data technique in studies with a broad observational instrument and a large database. Results show important gendered and cultural differences in POS use. Women tend to reproduce traditional gender role, being often more engaged in care functions with children and elders rather than in any other activity or with people of their same age group. Of particular concern is the gap on park use observed in women of ethnic minority groups. Assessing specific group needs on park use is particularly relevant attending to their multiple health and social benefits.

## Introduction

Public open spaces (POS) such as urban parks, open green spaces and squares, contribute to life quality in urban areas in many ways (Chiesura, 2004). Green spaces have stress-reduction and mental health benefits, as contact with nature has a number of restorative effects (Ulrich, 1984; Hartig et al., 1991; Ulrich et al., 1991; Hull and Michael, 1995; Kaplan, 1995; Hansmann et al., 2007; Collado and Staats, 2016). The largely free and accesible character of POS provides a setting for leisure activities and free opportunities for physical activity, which have been linked with multiple benefits to psychological and physical well-being, including weight management, controlling blood pressure, decreasing the risk of heart disease, strokes, breast cancer, and Type 2 diabetes (Godbey, 2009). Spending more time outdoors has also been linked with better health indicators because several indoor air pollutants and vitamin D deficiency, as a consequence of low sun exposure, are also associated with the pathogenesis of frequent chronic diseases (Viegi et al., 2004; Peterlik and Cross, 2005).

Additionally, from a psychological perspective, experiences in specific local places (e.g., public parks, squares, and markets) provide contexts for developing place-identity and might contribute to taste flow and well-being (Bonaiuto et al., 2016). POS are also essential for establishing social recognition and interaction, promoting friendship between neighbors, social cohesion, and a sense of community (Coley et al., 1997; Kuo et al., 1998; Cattell et al., 2008; Vargas and Merino, 2012). Cattell et al. (2008, p. 556) describe the beneficial properties of public spaces in community life: “Social interaction in public spaces, for example, can provide relief from daily routines, sustenance for people's sense of community, opportunities for sustaining bonding ties or making bridges, and can have a direct influence on wellbeing by raising people's spirits.” In a similar way, after pointing out the negative correlation between social cohesion and neighborhood insecurity, Vargas and Merino (2012, p. 172) claimed that “it is likely that perceptions of insecurity might decrease if children, youth, families and elder populations are integrated in the space with social activities creating social networks and a sense of community.” That is the crucial role of public spaces on social life.

Attending to the multiple benefits of POS on physical and psychosocial well-being, research has recently put more attention on questions related to environmental justice (Wolch et al., 2014). Using GIS-based measures, several studies have reported income and racial/ethnic disparities in access to recreation facilities, especially in the U.S. (Dahmann et al., 2010; Sister et al., 2010). On a recent review of the equity mapping literature on urban parks, Rigolon (2016) recently concluded that low socioeconomic status and ethnic minority communities have access to fewer parks, fewer park acres, and parks that are potentially more congested. Addressing social disparities in park provision not always require the creation of new public spaces, but also improving those that are underutilized.

Safety has been cited by both adolescents and adults, and in particular women, as one of the most important reasons for not using POS (Burgess et al., 1988; Valentine and Mckendrick, 1997; Molnar et al., 2004; Weir et al., 2006; Casper et al., 2013; Babey et al., 2015). In a review of qualitative research about park use, McCormack et al. (2010) found concerns as to the presence of “undesirable users” (e.g., drug users/dealers, homeless, and loiterers) also some park attributes related to injury safety (e.g., presence of glass, syringes, rocks, debris, heavy traffic) are also often mentioned as discouraging reasons for using public parks. This effect can be related to a disorder model about unsafety (Franklin et al., 2008), according to which both “social and physical incivilities are signs of lack of adherence to norms of public behavior” (Taylor and Hale, 1986, p. 154). Other studies have suggested that modifying park facilities could have a greater impact to increase park use than improving perceptions of park safety (Cohen et al., 2009; Lapham et al., 2016). Urban planners can play a key role in helping communities to have the same opportunities to access public parks. Assessing the type of users and activities that POS attract, can provide valuable information to identify existing disparities of access by certain specific groups. In contrast, as Sister et al. (2010) have stated, “the theoretical perspectives on social justice have seldom translated into practical methods and techniques applicable in the field, failing to provide specific tools for planners to assess, and address social disparities.”

Systematic observation has been proven effective in the analysis of natural contexts, respecting the maximum display of naturality (Anguera, 2003). Contrary to self-reports, systematic observation is a direct method that can provide objective information with strong internal validity and allows for the simultaneous generation of information about the physical and social environment where the activity is taking place (McKenzie and van der Mars, 2015). Recently, the analysis of park use with systematic observation has received considerable more attention, but because most of the studies have been conducted in the United States and focused on physical activity levels, important areas of interest remain still unclear.

Previous research consistently has shown a gender gap on park use, suggesting the existence of structural and cultural factors that influence women's leisure opportunities in an urban context (Scraton and Watson, 1998). More males than females tend to use public parks, being males more physically active than women (Evenson et al., 2016; Derose et al., 2018). According to Krenichyn (2004, p. 118), “women are underrepresented in urban parks and plazas and their absence is attributable to actual or perceived vulnerability to crime and threatening or sexually aggressive behavior, or that they use parks most often in the context of family and child-care activities.” Jackson and Henderson (1995, p. 48) also described women constrained in their leisure time “because of the social expectations (women are still primarily responsible for childcare in our society) and social controls (women make less money than men) associated with gender.” As a consequence, opportunities for leisure in public settings may be especially limited for women (Skogan and Maxfield, 1981; Hutchison, 1994; Perkins and Taylor, 1996).

The main goal of the present study is to offer a tool that can assist planners in addressing specific questions regarding park use. From a methodological point of view, our objectives are: (1) to present an observational instrument designed to record park use as naturally occurs in daily life and (2) to show an example of the possibilities that polar coordinates offer to analyse observational data. In this paper, we use this methodology from a gender perspective to explore gender disparities on park use in Barcelona, Catalonia (Spain). Barcelona is a city with a low and stable victimization index around 15%, basically referred to minor crimes and well-recognized urban public spaces (Valera and Guàrdia, 2014). Nevertheless, insecurity is usually defined by its citizens as one of the most important problems of the city, together with other topics also linked with fear of crime, such as cleanliness, immigration, vandalism, and poverty^1^. According to Subirats (2006), governance of the public space in Barcelona is today getting more complex as a result of economic, political, and social dynamics that implies greater job insecurity, more unemployed people on the streets, poverty, and ethnic diversification. A better understanding of how men and women use POS may ultimately lead to interventions to promote park use for all kinds of users and improve perceived safety on urban areas.

## Methods

### Design

We employed an N/F/M observational design (Blanco-Villaseñor et al., 2003; Anguera and Hernández-Mendo, 2013), where N refers to nomothetic (observing numerous POS and groups of people), F refers to intersessional follow-up (recording of numerous sessions) and M refers to multidimensional (analysis of multiple criteria included in the observational instrument).

### Participants

**Figure 1:**
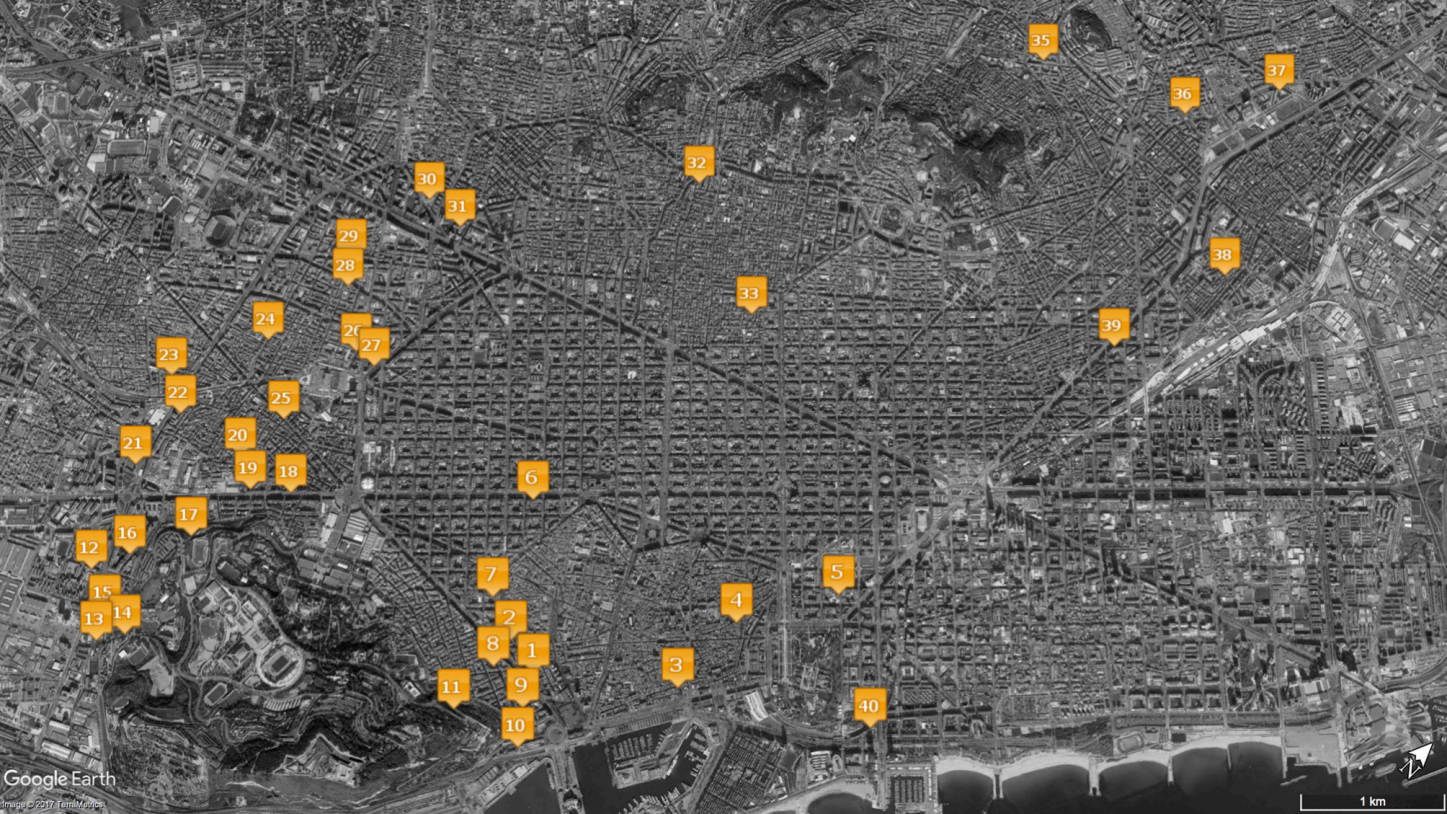
Location map for observed POS.

Forty POS distributed among all 10 districts of the city of Barcelona were analyzed (Figure 1). In order to have different levels of analysis, the sample included 20 POS in Sants-Montjuïc and 20 POS in nine different city districts. The election of the sample was oriented balancing the presence and absence of physical and social disorder signs. To organize observational data collection, 10 circular routes were defined, each one including 4 POS within <15 min walk or by public transportation. Exclusion criteria were (1) very small POS where the presence of an observer could easily produce reactivity and (2) an excessive distance between public spaces included on the same observational route. Final selection included open spaces (*n* = 2), open green spaces (*n* = 18), small town squares (*n* = 13), and large district parks (*n* = 7) across the city. When necessary, POS were divided into smaller targeted areas to facilitate systematic observation.

### Materials

#### Observational Instrument

An observational tool (EXOdES) was specifically created to assess park use and the environmental features of space where activity took place. EXOdES is an *ad hoc* instrument (Sánchez-Algarra and Anguera, 2013) based on the combination of field format and category systems, which permits recording co-occurrent behaviors regarding multiple criteria. This work was developed as part of a broader project, incluiding the development of new observational instrument to assess park use and the consequences of fear of crime on the activity patterns of public space. We conceptualized four different set of factors or macro-criteria: (1) contextual information (observer, date, observational period, public space, location/activity setting), (2) individual criteria (age, gender, and ethnicity of both people being alone and groups, size of groups, ethnic diversity of groups, poverty signs/homelessness), (3) activity criteria (main activity, dogs, vehicles, problematic uses, substances use signs, violence), and (4) environmental criteria (brightness, cleanness, visual control, green space maintenance, litter, graffiti). Category systems were defined for those criteria with limited options (e.g., gender, age, and race/ethnicity) and catalogs were created for those criteria with unlimited possibilities (i.e., type of vehicles and the main activity sports) that could be extended in case of observing new responses not previously considered. An earlier pilot and more details about the observational instrument and procedure can be found in previous works (Pérez-Tejera et al., 2011; Pérez Tejera, 2012; Valera et al., 2018). Six criteria of the observational system were selected for the present study to describe park use: time of day, age group, race/ethnicity, location, activity, and vehicles (Table 1). Environmental factors were excluded for a question of space and other relevant criteria regarding park use -problematic uses, substances use signs, violence, poverty signs- were also excluded for being infrequent, although their park use implications can be explored in the future.

**Table 1:** EXOdES park use observation instrument.

**Criterion**	**Code**	**Description**
Observation period (TIME)	1011	10:00–11:00
	1112	11:00–12:00
	1213	12:00–13:00
	1314	13:00–14:00
	1617	16:00–17:00
	1718	17:00–18:00
	1819	18:00–19:00
	1920	19:00–20:00
Gender (GEND)	GFEM	Female group
	GMAS	Male group
	GMIX	Mixed gendered group
Age (AGE)	GCHI	Children
	GYOU	Youths
	GADU	Adults
	GELD	Elders
	ADEL	Adults with elders
	CHYO	Children and youths
	CYAE	Children and/or youths with adults and/or elders
Race/ethnicity (ETHN)	WHIT	White
	LATI	Latin/Caribbean
	ARAB	Arab
	ASIA	Asian
	AFRI	African
Activity setting (SETT)	BENC	Benches or similar
	PLAG	Playgrounds
	OPEN	Open spaces
	COUR	Sport courts
	GREE	Green areas
	SOTH	Other settings
Activity (USE)	SITT	Enjoying the scenery, chatting or relaxing
	PLAY	Playing
	WALK	Walking
	FOOT	Playing football
	PETA	Playing boules
	OSPO	Playing other sports
	PICK	Picnicking
Vehicles (VEHI)	NOVE	No vehicles
	BICY	Bicycle
	SKAT	Skate or roller skater
	BABY	Stroller
	WHEE	Wheelchair
	DRIV	Motorized vehicles

#### Observers

Eight observers and two digital recorders were contracted half-time by the City Council of Barcelona and coordinated by the researchers. Training consisted of in-class and field-based training and occurred over the course of 1 month. In-class training provided an overview of the study purpose, data collection materials, park observation protocols, and EXOdES training with photographs. Field-based training consisted of on-site visits to each park to review its location and to practice the data collection with EXOdES under investigator supervision. Observers participated in the elaboration of detailed maps of each park identifying all targeted areas within each (e.g., football field, play-ground equipment, and open space). The control of quality of data has been done through kappa Cohen's coefficient, that has been satisfactory, exceeding 80%. Also, correlation coefficient is higher than 0.80.

### Procedure

Systematic records were performed between September 2010 and December 2010. All POS were visited 8 times per day (observation period): 10:00–11:00, 11:00–12:00, 12:00–13:00, 13:00–14:00, 16:00–17:00, 17:00–18:00, 18:00–19:00, and 19:00–20:00. After assuring high levels of inter-rater reliability during training, observations were conducted by 1 observer. Every weekday during the study period every observer was assigned to one of 10 routes including 4 POS, in a morning (from 10:00 to 14:00) or afternoon turn (from 16:00 to 20:00). Each observational session was defined as a 45-min observational period. After the first observational session, the observer moved to the next POS of the route and started a new 45-min observational session until complete the assigned route. With this procedure, short observation sessions were ensured reducing the risk of observer fatigue and reactance (Hoeben et al., 2018). At the end of the study, every POS was observed at 8 different observational periods, a median of 5 different days, by at least 3 different observers and 3 different weekdays, to diminish some bias. Observations were conducted only during good weather. When special events took place in the POS, observational sessions were rescheduled on the same weekday in the following weeks.

During each observational session, observational scans of target areas were performed periodically to obtain information about park use. A scan is a single observation or visual sweep from left to right across the target area. All individuals or groups observed in each location during a 45-min observational period were recorded naturally. In the case of individuals, age group (i.e., children, teens, adults, and elders), and race/ethinicity (i.e., White, Latin, Arab, Asian, and African) were recorded. The make-up of the groups were recorded accordingly: size of the group (i.e., 2, 3–5, 6–10, and 10–20), gender composition (i.e., men, women, mostly men, mostly women, and equally mixed), age (i.e., children, teens, adults, elders, children with teens, adults with elders, and children/teens with adults/elders), and ethnicity using the same taxonomy for individuals. Aditionally, groups were also classified regarding their ethnic homogenity (i.e., Whites, mostly whites, equally mixed, mostly non-whites, and non-whites). The activity setting or target area where people were observed (e.g., sport court, playground, and open space), the activity (e.g., play, sports, and walk) and the presence or absence of vehicles (e.g., no vehicles, skate, and stroller) were also recorded. Thus, each individual or group using the space during an observational session were recorded as a configuration, providing information regarding the co-occurrent multidimensional criteria of the observational instrument.

This research was carried out in accordance with the Declaration of Helsinki. A review by an ethics committee and written informed consents were not required in this study as: (a) it involved the observation of people in public places where individuals or groups targeted for observation had no reasonable expectation of privacy; (b) it did not include any intervention staged by the researcher or direct interaction with the individuals or groups; and (c) it did not comprise collecting personal information disseminated through photographic, film or video footage in the research results.

### Data Analysis

Configurations recorded in all 40 POS were compiled as a global data set. We estimated the number of people observed counting for the number of individuals and groups of two people. When the size of groups was 3–5, 6–10, or 10–20, the number was estimated based on modal values. Regarding gender in groups, we considered that 0.75, 0.5 and 0.25% were women when the gender composition was coded as mostly women, equally mixed and mostly men, respectively.

Thus, information on the behavior of more than 18,000 groups were analyzed to search for regular structures hidden in data set according to gender. Prospective and retrospective multievent sequential analysis, from lag −5 to lag +5, were performed using GSEQ 5.1 (Bakeman and Quera, 1995, 2011). We used a simplified gender composition category -groups of males only (GMAS), females only (GFEM), and gendered mixed (GMIX)- as target behaviors, considering the rest of categories in the observational instrument as given criteria. Thus, several polar coordinate analysis were performed with HOISAN (Hernández-Mendo et al., 2012) to create maps with all possible interrelations between gender composition of observed groups and all categories of the field format.

**Figure 2:**
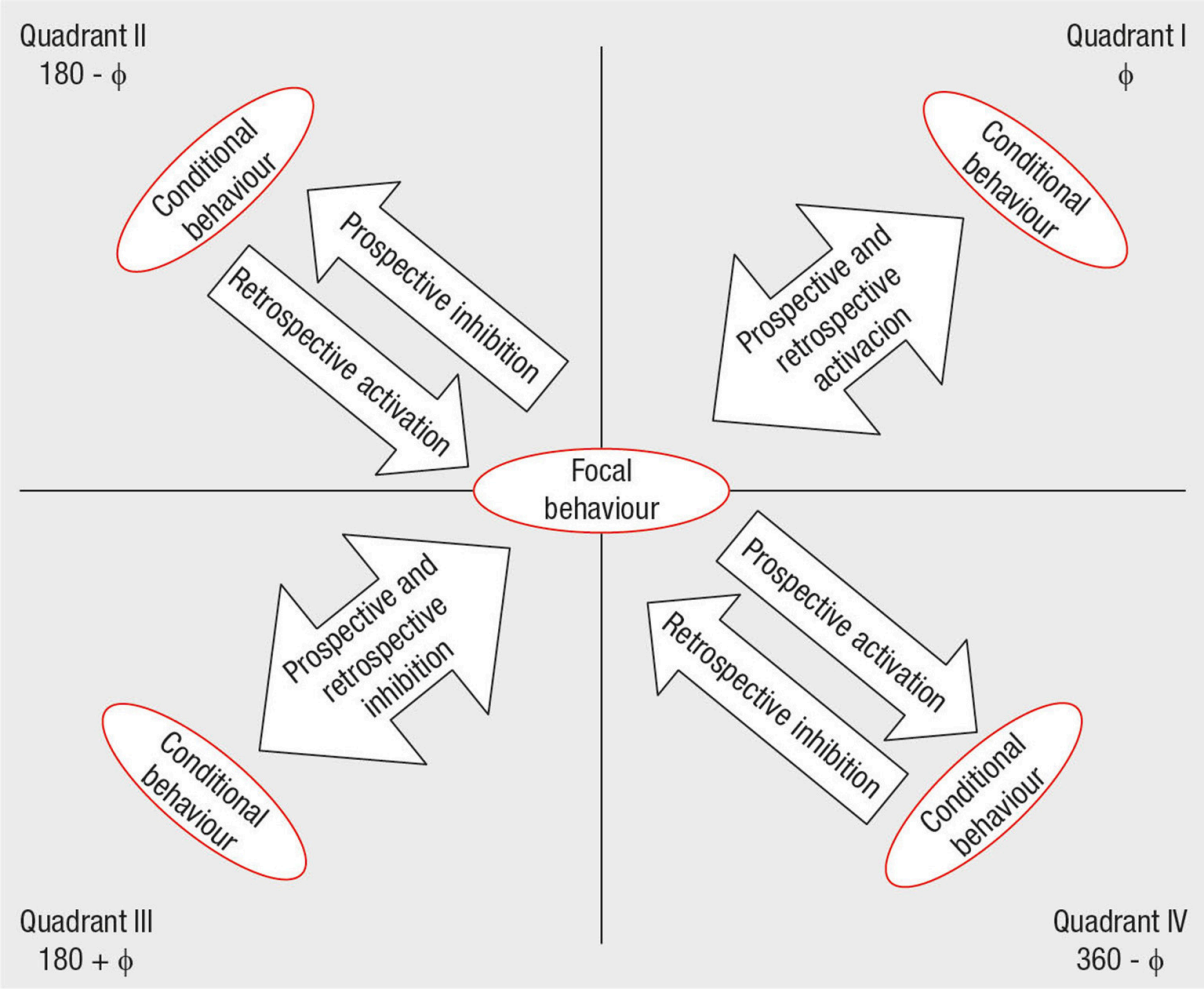
Relationships between focal and target behaviors in a polar coordinate map (Extracted from Aragón et al., 2016, p. 5).

Polar coordinate is a data reduction technique based on the *Z*_*sum*_ statistic, which was introduced by Cochran (1954), developed by Sackett (1980), and optimized by Gorospe and Anguera (2000). Standardized Z statistics derived from adjusted residuals (Bakeman, 1978) were used to compute prospective and retrospective *Z*_*sum*_ statistics. These values are then used to build maps showing the relationships between a focal behavior and one or more conditional behaviors. These relationships are considered significant (*p* < 0.05) when the vector length is >1.96 (excitatory) or < −1.96 (inhibitory). Each quadrant shows the type of relationship between the focal behavior and the corresponding conditional behavior as follows (Figure 2): Quadrant I: prospective and retrospective activation; Quadrant II: prospective inhibition and retrospective activation; Quadrant III: prospective and retrospective inhibition; and Quadrant IV: prospective activation and retrospective inhibition. Although this technique was specifically developed for use in sport research (Gorospe and Anguera, 2000; Perea et al., 2012; Aragón et al., 2016; Castañer et al., 2016; Tarragó et al., 2016), it has been also useful in other fields (Anguera et al., 2003; Herrero Nivela and Pleguezuelos Saavedra, 2008; Santoyo et al., 2017). To our best knowledge, it is the first time that it was applied to analyse daily life interactions in public spaces.

## Results

Research staff completed a total of 1,505 observational sessions made on 67 different days. During the study period, we estimate that 75,853 people (55 males vs. 45% females) were using POS during observational periods. Specifically, 16,209 people were observed as being alone (66 males vs. 34% females) and we estimate that 59,644 people were recorded in groups (53 males vs. 47% females). The complete observed categories among males only (GMAS), females only (GFEM), and gendered mixed groups (GMIX) are shown in Table 2. For all studied criteria, the chi-square for differences among gender groups was significant at the 0.001 level.

**Table 2:** Observed frequency of analyzed criteria by gender composition.

		**Male groups**		**Female groups**		**Mixed groups**		**Total**	
		***N***	**%**	***N***	**%**	***N***	**%**	***N***	**%**
**OBSERVATION PERIOD**									
1011		373	6.0%	317	5.1%	349	6.0%	1,039	5.7%
1112		670	10.7%	631	10.1%	557	9.6%	1,858	10.2%
1213		751	12.0%	755	12.1%	629	10.9%	2,135	11.7%
1314		709	11.3%	621	10.0%	554	9.6%	1,884	10.3%
1617		632	10.1%	436	7.0%	495	8.6%	1,563	8.5%
1718		1,094	17.5%	1,507	24.1%	980	17.0%	3,581	19.6%
1819		1,078	17.2%	1,224	19.6%	1,137	19.7%	3,439	18.8%
1920		949	15.2%	749	12.0%	1,071	18.6%	2,769	15.2%
XSQ	257.62	df = 14	*p* = < 0.01					
**AGE**									
GCHI		541	8.7%	222	3.6%	394	6.8%	1,157	6.3%
GYOU		1,433	22.9%	695	11.1%	1,168	20.2%	3,296	18.0%
GADU		1,689	27.0%	925	14.8%	1,623	28.1%	4,237	23.2%
GELD		670	10.7%	428	6.9%	782	13.5%	1,880	10.3%
ADEL		170	2.7%	413	6.6%	325	5.6%	908	5.0%
CHYO		80	1.3%	67	1.1%	65	1.2%	212	1.2%
CYAE		1,666	26.7%	3,490	55.9%	1,415	24.6%	6,571	36.0%
XSQ	1,970.5	df = 12	*p* = < 0.01					
**RACE/ETHNICITY**									
WHIT		4,368	71.5%	4,851	79.0%	4,734	83.7%	13,953	77.9%
LATI		821	13.4%	780	12.7%	591	10.5%	2,192	12.3%
ARAB		386	6.3%	155	2.5%	91	1.6%	632	3.5%
ASIA		382	6.2%	299	4.9%	198	3.5%	879	4.9%
AFRI		156	2.6%	56	0.9%	42	0.7%	254	1.4%
XSQ	421.95	df = 8	*p* = < 0.01					
**ACTIVITY SETTING**									
BENC		2,620	41.9%	2,595	41.6%	2,865	49.6%	8,080	44.2%
PLAG		900	14.4%	1,927	30.9%	782	13.6%	3,609	19.8%
OPEN		1,733	27.7%	1,340	21.5%	1,547	26.8%	4,620	25.3%
COUR		578	9.2%	85	1.4%	129	2.2%	792	4.3%
GREE		197	3.2%	146	2.3%	237	4.1%	580	3.2%
SOTH		227	3.6%	142	2.3%	212	3.7%	581	3.2%
XSQ	1,293.17	df = 10	*p* = < 0.01					
**ACTIVITY**									
SITT		3,453	58.9%	3,497	56.3%	3,889	69.3%	10,839	61.3%
PLAY		950	16.2%	1,826	29.5%	794	14.1%	3,570	20.2%
WALK		331	5.6%	623	10.0%	646	11.5%	1,600	9.0%
FOOT		427	7.3%	39	0.6%	66	1.2%	532	3.0%
PETA		163	2.8%	13	0.2%	32	0.6%	208	1.2%
OSPO		403	6.8%	72	1.2%	89	1.6%	564	3.2%
PICK		140	2.4%	139	2.2%	98	1.7%	377	2.1%
XSQ	1,729.23	df = 12	*p* = < 0.01					
**VEHICLES**									
NOVE		5,125	82.0%	4,345	69.6%	4,696	81.4%	14,166	77.5%
BICY		275	4.4%	116	1.9%	150	2.6%	541	3.0%
SKAT		186	3.0%	67	1.1%	55	1.0%	308	1.7%
BABY		376	6.0%	1,506	24.1%	574	10.0%	2,456	13.5%
WHEE		96	1.5%	188	3.0%	203	3.5%	487	2.7%
DRIV		195	3.1%	17	0.3%	88	1.5%	300	1.7%
XSQ	1,285.15	df = 10	*p* = < 0.01					

*Only those categories accounting for more than 1.0% have been included; those with less than 1.0% were aggregated in other recoded or eliminated of the analysis*.

Table 3 shows the polar coordinates analysis numerical result, considering as focal behavior GMAS, GFEM, and GMIX. It includes the following information: name of the conditional behavior, quadrant, prospective and retrospective *Z*_*sum*_, radius, and angle. The polar coordinate maps offer a visual representation of the statistically significant associations (activation or inhibition) between focal and conditional behaviors. In the present study, only significant relations between focal and conditional behaviors are presented. The association is shown both quantitatively (length of vector) and qualitatively (quadrant I, II, III, or IV). We have structured results into sections organized by the different 6 target criteria in EXOdES that have been analyzed.

**Table 3:** Polar coordinate analysis of studied criteria considering gender composition the focal behavior.

	**Male groups (GMAS)**					**Female groups (GFEM)**					**Mixed groups (GMIX)**				
**Code**	**Quadr**.	**Prosp**.	**Retrosp**.	**Radius**	**Angle**	**Quadr**.	**Prosp**.	**Retrosp**.	**Radius**	**Angle**	**Quadr**.	**Prosp**.	**Retrosp**.	**Radius**	**Angle**
TIME_1011	I	4.75	2.58	5.41 (*)	28.45	III	−9.07	−4.71	10.23 (*)	207.45	I	4.4	2.17	4.91 (*)	26.28
TIME_1112	I	3.51	4.31	5.56 (*)	50.84	IV	1.85	−0.49	1.91	345.09	III	−5.47	−3.9	6.72 (*)	215.52
TIME_1213	I	1.37	1.44	1.99 (*)	46.46	I	2.34	2.52	3.44 (*)	47.16	III	−3.78	−4.05	5.54 (*)	226.93
TIME_1314	I	7.1	7.38	10.24 (*)	46.1	III	−3.16	−3.13	4.45 (*)	224.76	III	−4.03	−4.34	5.93 (*)	227.11
TIME_1617	I	11	12.08	16.34 (*)	47.68	III	−11.98	−10.63	16.02 (*)	221.59	IV	0.99	−1.48	1.78	303.85
TIME_1718	III	−10.95	−13.63	17.48 (*)	231.21	I	24.84	24.54	34.92 (*)	44.66	III	−14.16	−11.13	18.01 (*)	218.17
TIME_1819	III	−10.04	−9.21	13.63 (*)	222.51	I	5.03	3.51	6.13 (*)	34.91	I	5.13	5.82	7.76 (*)	48.6
TIME_1920	I	0.98	2.7	2.87 (*)	69.96	III	−18.26	−18.92	26.29 (*)	226.02	I	17.61	16.54	24.16 (*)	43.21
AGE_GCHI	I	10.88	10.74	15.29 (*)	44.62	III	−10.08	−8.52	13.2 (*)	220.2	III	−0.75	−2.12	2.25 (*)	250.52
AGE_GYOU	I	15.43	14.55	21.21 (*)	43.31	III	−22.69	−22.85	32.2 (*)	225.2	I	7.7	8.8	11.69 (*)	48.83
AGE_GADU	I	20.57	22.97	30.83 (*)	48.15	III	−32.83	−33.54	46.93 (*)	225.61	I	12.96	11.28	17.18 (*)	41.05
AGE_GELD	I	3.4	5.62	6.57 (*)	58.84	III	−11.82	−13.99	18.32 (*)	229.81	I	8.81	8.73	12.41 (*)	44.75
AGE_ADEL	III	−8.74	−9.33	12.78 (*)	226.87	I	5.23	4.5	6.9 (*)	40.74	I	3.59	4.83	6.02 (*)	53.39
AGE_CHYO	I	1.8	1.74	2.5 (*)	44.06	III	−1.39	−4.06	4.29 (*)	251.09	II	−0.41	2.42	2.46 (*)	99.63
AGE_CYAE	III	−33.95	−36.27	49.68 (*)	226.89	I	56.2	58.38	81.04 (*)	46.09	III	−23.49	−23.42	33.17 (*)	224.92
ETHN_WHIT	III	−21.17	−17.91	27.73 (*)	220.23	I	4.8	2.83	5.58 (*)	30.51	I	16.75	15.38	22.74 (*)	42.55
ETHN_LATI	I	7.45	3.97	8.44 (*)	28.07	I	4.62	5.17	6.94 (*)	48.19	III	−12.35	−9.33	15.48 (*)	217.06
ETHN_ARAB	I	16.83	14.97	22.53 (*)	41.66	III	−9.14	−6.82	11.4 (*)	216.74	III	−7.88	−8.32	11.46 (*)	226.53
ETHN_ASIA	I	6.13	5.8	8.44 (*)	43.43	III	−3.19	−1.78	3.65 (*)	209.17	III	−3.01	−4.11	5.09 (*)	233.71
ETHN_AFRI	I	16.05	17.46	23.71 (*)	47.42	III	−8.99	−9.73	13.25 (*)	227.26	III	−7.22	−7.88	10.69 (*)	227.49
SETT_PLAG	III	−20.76	−25.7	33.03 (*)	231.07	I	43.7	45.38	63 (*)	46.08	III	−23.45	−20.08	30.87 (*)	220.58
SETT_GREE	III	−3.6	−3.72	5.17 (*)	225.95	III	−6.03	−5.59	8.22 (*)	222.86	I	9.84	9.49	13.67 (*)	43.98
SETT_BENC	I	1.67	3.12	3.54 (*)	61.78	III	−11.48	−13.11	17.43 (*)	228.78	I	10.04	10.2	14.31 (*)	45.44
SETT_COUR	I	28.94	28.71	40.77 (*)	44.77	III	−18.95	−15.14	24.26 (*)	218.63	III	−10.22	−13.84	17.21 (*)	233.55
SETT_OPEN	I	4.44	5.89	7.38 (*)	52.98	III	−11.35	−11.85	16.4 (*)	226.24	I	7.07	6.08	9.32 (*)	40.7
SETT_SOTH	I	1.52	4.9	5.13 (*)	72.8	III	−5.79	−8.27	10.1 (*)	235.03	I	4.37	3.44	5.57 (*)	38.21
USE_SITT	I	3.56	5.06	6.19 (*)	54.83	III	−20.81	−21.43	29.88 (*)	225.84	I	17.73	16.8	24.42 (*)	43.45
USE_PLAY	III	−15.51	−21.08	26.17 (*)	233.66	I	39.44	40.33	56.41 (*)	45.63	III	−24.7	−19.87	31.7 (*)	218.82
USE_WALK	III	−12.72	−8.29	15.19 (*)	213.09	IV	0.48	−2.16	2.21 (*)	282.52	I	12.47	10.62	16.38 (*)	40.41
USE_OSPO	I	17.9	16.97	24.66 (*)	43.48	III	−12.92	−9.73	16.17 (*)	216.98	III	−4.96	−7.27	8.8 (*)	235.66
USE_FOOT	I	18.97	17.24	25.63 (*)	42.26	III	−10.03	−7.84	12.73 (*)	218.01	III	−9.02	−9.46	13.07 (*)	226.37
USE_PICK	I	2.32	3.05	3.84 (*)	52.77	III	−0.81	−1.2	1.45	235.97	III	−1.54	−1.87	2.42 (*)	230.55
USE_PETA	I	17.59	20.59	27.08 (*)	49.49	III	−10.8	−11.61	15.86 (*)	227.07	III	−6.82	−9	11.29 (*)	232.86
VEHI_NOVE	I	18.18	20.28	27.23 (*)	48.12	III	−31.11	−30.98	43.91 (*)	224.88	I	13.23	10.94	17.17 (*)	39.59
VEHI_BICY	I	5.3	6.18	8.14 (*)	49.41	III	−7.4	−6.51	9.85 (*)	221.32	I	2.16	0.33	2.18 (*)	8.73
VEHI_BABY	III	−30.86	−32.67	44.94 (*)	226.63	I	47.58	46.48	66.52 (*)	44.33	III	−17.11	−14.12	22.18 (*)	219.54
VEHI_WHEE	III	−7.08	−8.62	11.16 (*)	230.58	I	3.93	3.08	4.99 (*)	38.09	I	3.22	5.65	6.5 (*)	60.28
VEHI_DRIV	I	14.11	12.79	19.05 (*)	42.2	III	−12.33	−10.92	16.46 (*)	221.53	III	−1.83	−1.91	2.64 (*)	226.1
VEHI_SKAT	I	7.77	7.24	10.62 (*)	42.99	III	−4.37	−3.23	5.44 (*)	216.47	III	−3.47	−4.09	5.36 (*)	229.7

* *Significant relationships (p < 0.05) between the focal behavior and conditional behaviors*.

### Time of Day

**Figure 3:**
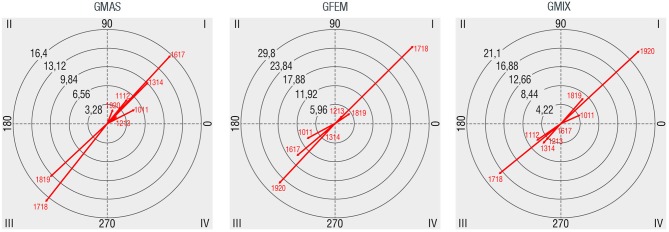
Polar coordinate maps considering observational periods as target behavior (1011: 10:00–11:00, 1112: 11:00–12:00, 1213: 12:00–13:00, 1314: 13:00–14:00, 1617: 16:00–17:00, 1718: 17:00–18:00, 1819: 18:00–19:00, 1920: 19:00–20:00).

Studied POS have more capacity to attract groups of people during the afternoon, especially from 17:00 to 20:00. As shown in Table 2, during this observational period, 53.6% of groups were observed. In Figure 3, relationships between gender composition and observational periods are shown. Male groups have mutually inhibitory relationships with 17:00–18:00 (1718) and 18:00–19:00 (1819), also mutually excitatory relationships with the rest of observational periods. Contrary to men groups, female groups present mutually excitatory relationships with 12:00–13:00 (1213), 18:00–19:00, and particularly significant with 17:00–18:00, coinciding with the moment when children finish school in Spain. Regarding mixed groups, mutually excitatory relationships are found with 10:00–11:00 (1011), 18:00–19:00, and particularly stronger with 19:00–20:00 (1920).

### Age

**Figure 4:**
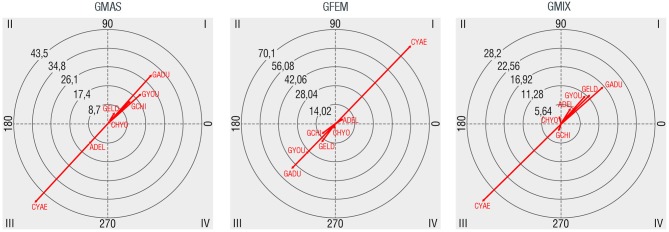
Polar coordinate maps considering age composition of groups as target behavior (GCHI: children, GYOU: youths, GADU: adults, GELD: elders, ADEL: adults with elders, CHYO: children with youths, CYAE: children and/or youths with adults and/or elders).

The most frequent composition group observed (36.0%) was that formed by children, youth or both, accompanied by adults, older adults or both (CYAE). This category comprises of different forms of child and youth care. After that, the most common groups were adults (23.2%), youths (18.05%), elders (10.3%), children (6.3%), adults with elders (5.0%), and children with youths (1.2%). In the second polar coordinate map, the relationship between gender and age groups are shown. As we can see in Figure 4, male groups have mutually excitatory relations with all composition groups, except with groups of adults and older adults (ADEL), and particularly groups of children and/or youths with adults and/or older adults (CYAE), both with mutually inhibitory relations. Groups of adults with older adults, but particularly groups of children and/or youths supervised by adults and/or older adults are the only composition groups that are found to be mutually activated with groups of females. Regarding mixed groups, mutually excitatory associations are found with groups of youths (GYOU), adults (GADU), elders (GELD), and groups of adults with elders. Mixed gendered groups also have mutually inhibitory relationships with groups of children only (GCHI) and groups of children and/or youths supervised by adults and/or older adults.

### Race/Ethnicity

**Figure 5:**
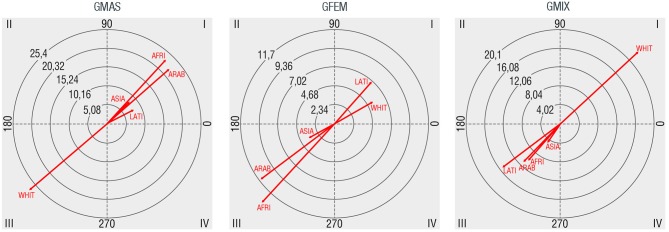
Polar coordinate maps considering race/ethnicity composition group as target behavior (WHIT: whites, LATI: latins, ARAB: arabs, ASIA: asians, AFRI: africans).

Regarding race/ethnicity, most of the observed groups are Whites (77.9%), followed by Latins (12.3%), Asians (4.9%), Arabs (3.5%), and Africans (1.4%). These results are coherent with the heterogeneity of residents in the city of Barcelona, as according to the census 16.6% of its population is foreign, that being Europeans, Latins, and Asians the more common origins. The groups ethnically heterogeneous, those where whites and other minority groups are mixed, represent the 7.5% of observed groups. In Figure 5, we show the relationships that have been found between gender and race/ethnicity. Male groups have mutually excitatory connections with all minority groups, particularly stronger with Africans (AFRI) and Arabs (ARAB). Whites (WHIT) is the only category with which male groups establish a strong mutually inhibitory relationship. Female groups, on the other hand, have mutually excitatory relations with Latins (LATI) and Whites, also mutually inhibitory relationships with Asians (ASIA) and particularly Arabs and Africans. Mixed gendered groups have a mutually excitatory relationship only with Whites and mutually inhibitory relations with the rest of minority groups.

### Activity Setting

**Figure 6:**
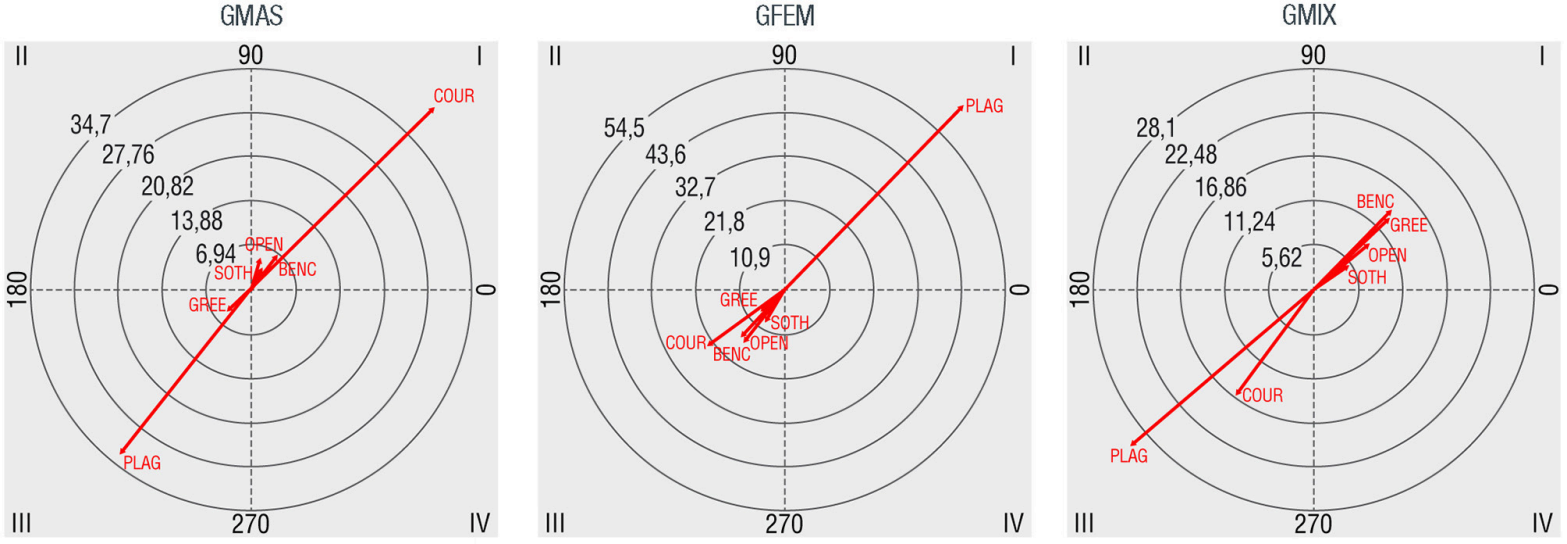
Polar coordinate maps considering location where the activity occurs as target behavior (BENC: benches or similar, PLAG: playground, OPEN: open space, COUR: sport courts, GREE: green areas, SOTH: other settings such as fountains or parking lots).

The most heavily used activity settings were those where people can sit such as benches, little walls or stairs (44.2%), followed by open spaces (25.3%), playgrounds (19.8%), sport courts (4.3%), and green areas (3.2%). Figure 6 examines the kind of relations established between activity setting and gender group. Male groups have mutually excitatory connections with benches, little walls or stairs (BENC), open spaces (OPEN), and particularly with sport courts (COUR). Green spaces (GREE) and specially playgrounds (PLAG) have mutually inhibitory connections with male groups. On the contrary, playground is the only activity setting that has a mutually excitatory relationship with female groups. Regarding mixed groups, they have mutually excitatory connections with all activity settings except with sport courts and playgrounds.

### Activity

**Figure 7:**
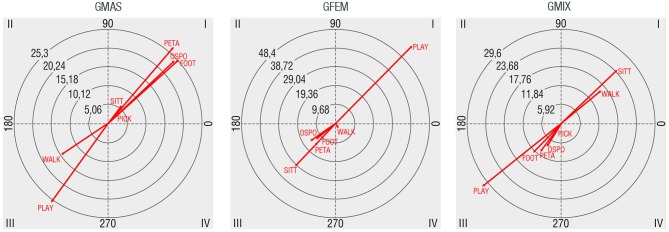
Polar coordinate maps considering activity as target behavior (SITT: just enjoying the scenery, chatting or relaxing, PLAY playing, WALK: walking, FOOT: playing football, PETA: playing boules, OSPO: playing other sports such as volleyball, PICK: picnicking).

The most common activities observed were sitting or chatting (61.3%), followed by playing (20.2%), walking (9.0%), playing sports (7.4%), and picnicking (2.1%). Most frequently observed sports were football (3.0%) and boules (1.2%). Relationships that have been detected between the activity and gender are shown in Figure 7. Male groups have mutually excitatory relationships with sitting/chatting (SITT) and picnicking (PICK), but the strongest relationships are established with playing sports as football (FOOT), boules (PETA) or others (OSPO). Regarding female groups, the only activity that is mutually activated is that related to game (PLAY) activities. In mixed gendered groups, activities that are found mutually activated are sitting/chatting and walking (WALK).

### Vehicles

**Figure 8:**
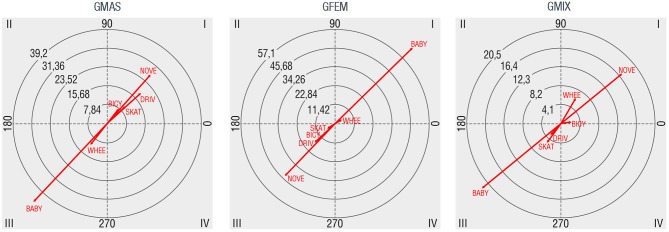
Polar coordinate maps considering vehicles use as target behavior (NOVE: vehicle absence, BICY: bicycle, SKAT: skate or roller skates, BABY: stroller, WHEE: wheelchair, DRIV: motorized vehicles such as cars or motorcycles).

The analysis of vehicles is a complementary way of describing park use. From observed groups, 22.4% were carrying some type of vehicle, stroller being the most frequent (13.5%) followed by bicycles (3.0%), wheel chairs (2.7%), skates or roller skaters (1.7%), and other motorized vehicles as cars or motorcycle (1.7%). Figure 8 shows the type of relationships found between gender groups and vehicles. Male groups have mutually excitatory relations with no vehicles (NOVE), motorized vehicles (DRIV), skates (SKAT) and bicycles (BICY), and mutually inhibitory relationships with wheelchairs (WHEE) and particularly with baby carriages (BABY). Female groups have mutually excitatory relations with strollers and wheelchairs, also mutually inhibitory relationships with the rest of vehicles. Finally, mixed gendered groups have mutually excitatory relationships with bicycles, wheelchairs and particularly with no vehicles.

## Discussion

Public open spaces (POS) play a critical role in urban areas offering free opportunities for leisure and physical activity. They also help to increase social recognition and interaction with neighbors, which is the basis to improve social cohesion, trust, and perception of safety. Nevertheless, few studies have used systematic observation to analyse activity patterns on POS except for some recent studies on outdoor physical activity mainly in the United States.

We have conducted a systematic observation study over 3 months, observing 40 POS distributed across all 10 districts of Barcelona to assess gendered differences on park use. An *ad hoc* observational instrument (EXOdES) was used to record sociodemographic characteristics of park users and their activities. In this paper, we estimated numbers of people using POS and analyzed gendered differences on several criteria: time of day, age group, race/ethnicity, activity setting, activity, and vehicles.

According to the census, less males than females (47 vs. 53%) live in Barcelona. Nevertheless, more males than females (55 vs. 45%) regardless of age group were seen using POS. The difference was particularly higher in individuals (66 vs. 34%) rather than in groups, where numbers were more similar (53 vs. 47%). This result is consistent with several previous studies in other geographic areas. In a recent review of observational studies measuring physical activity levels on park users, Evenson et al. (2016) found that in 20 studies more males than females were observed using public parks, ranging from 51 to 67%, while just three of them reported fewer males or no gender differences. Thus, a considerable higher proportion of females were seen in the present study when compared to several of those included in Evenson's review. Regarding groups of the same age, more adults, and adolescents were seen than older adults and children, which is also consistent with literature. However, the most frequent mixed age group composition in our study was adults and/or older adults with children and/or youngs (36%), thus a considerable proportion of elders and children were observed using POS.

To particularly assess differences in the way that men and women use public space, data regarding to more than 18,000 groups of people have been analyzed considering their gender composition (males only, females only and mixed gendered groups). Using multievent sequential and polar coordinate analysis, several hidden patterns in dataset have been identified. Groups of women were more likely to use POS between 17:00 and 18:00 (after children finish school) rather than at other times of the day. They were particularly infrequent between 19:00 and 20:00 unless they were with other men.

Contrary to men, women were more frequently involved in groups with other children, adolescents, and elders, rather than with other women of their same age group. Women were also more likely to be close to playgrounds areas where they could supervise children, to be engaged in play activities with them, and to be seen with strollers, rather than any other amenities, activities or vehicles. All these results show that outdoor leisure of women is largely centered around traditional family roles as they spend more time with children, elderly or disabled relatives (Hutchison, 1994; Kavanagh et al., 2006). These care functions were rarely seen in groups of males and mixed groups, according to codes that were mutually activated and inhibited in polar coordinate maps.

On the other hand, male groups were more likely to be observed at any time of day except from 17:00 to 19:00. Men were more often found amongst people of the same age group, using considerably more activity settings than women (e.g., sport courts, benches, and open spaces) and performing activities such as enjoying the scenery, chatting, relaxing, and picnicking. Consistently with literature, a higher proportion of men were engaged in moderate and vigorous activities, as playing football, basketball, boules, skating, or bicycling (McKenzie et al., 2006; Reed et al., 2008; Parra et al., 2010). From a health perspective, women's constraints on outdoor physical activity is of particular concern due to the important benefits on health indicators. A policy challenge is how to engage more women in sports while simultaneously supplying other sources of care for their young children (Cohen et al., 2007).

Two important questions arise when considering the role of race/ethinicity on park use. Firstly, a considerable fewer proportion of Asian, Arab, and African women were seen compared with groups of men of the same ethnic group. When female groups were observed, they were more likely to be Whites or Latinas rather than any other origin. However, groups of men have mutually excitatory relationships with all minority groups and an inhibitory relation just with Whites. These results reflect the exclusion of public space that many women from minority ethnic groups experience, which is coherent with previous ethnographies conducted in Barcelona (Garcia-Ramon et al., 2004; Ortiz et al., 2004). Research has shown that women from minority ethnic groups may have some specific constraints for park use, including a higher fear of sexual and racial attack, differences in roles and rights by gender as a result of more patriarchal structures (Ho et al., 2005), restrictions related to matters of honor especially on Muslim women (Peters, 2011), a socio-economic situation that decreases the importance of leisure pursuits and a “fear of dogs” mainly associated with religious reasons (Rishbeth, 2001). Secondly, while POS was frequented by a range of different ethnical groups, just 7.5% of observed groups were seen having contact between them. Ethic segregation may be highly functional for some groups when segregation is voluntary. Ethnic minorities “frequently want to be together in order to enjoy mutual support, rebuild family and neighborhood networks, and maintain their languages and cultures” (Castles, 1993). However, more efforts are needed to encourage informal social contact in POS between different ethnic groups. Promoting heterogeneity, tolerance and inter-ethnic understanding have also been linked with social cohesion and perception of safety (Vargas and Merino, 2012).

In the light of above exposed, a final reflection about the social quality of POS could be made. One of the most important consequences of fear of crime is the withdrawal of people from public spaces, especially vulnerable social groups (Jackson, 2011; Rader et al., 2012; Shippee, 2012). Fear of crime can make people prisoners at their own home (Hale, 1996). People who are afraid of being criminally victimized tend to stay more at home, limiting their social and cultural activities, reducing the quality of life, and eroding social life. Additionally, limiting one's movement to safe places at safe times may have a feedback loop: limiting social interaction also increases fear in its turn (Liska et al., 1988), whereas, experiencing ethnic and social variety regularly may help to develop a sense of familiarity with strangers, reduce intolerance and increase social cohesion, perceptions of safety and well-being (Kazmierczak, 2013). From an urban ecological perspective (Saunders, 2001) social diversity has a great relevance on urban social management. For instance, Hristova et al. (2016) considers “brokerage” (or social connectivity), “serendipity,”, “entropy,” and “homogeneity” as mesures of social diversity. Indeed, POS should provoque spontaneous and unexpected social encounters, as well as those planned and trusted. Because POS are the main scenarios for urban social life, contact with strangers–viewed as an opportunity, not as a risk–should be psychosocially enriching, and a tool for pomoting social cohesion. In many cities, as we have also seen in Barcelona, too many POS are places appropiated by specific social groups in specific periods of time. This is particullary dramatical when we have analised gender patterns of occupation specially related to female traditional roles as well as cultural ethnic differences. Thus, considered, it is only a matter of time that POS will end up loosing their social relevance in favor of other more controlled and safer places. Conversely, a higher interest in promoting social diversity in a perceived safety environments could break off this tendency, now broadly extended in many urban environments (Low, 2003).

Some limitations of this study have been identified. Probably, the most important one was that observations were conducted only on weekdays from September to December. Thus, any conclusion about the activity patterns in studied public spaces should be restricted to this observational period. It would be essential to examine POS during weekends, as gendered patterns of public space use may be different, also during other times of the year to identify seasonal changes on park use. A second limitation included the sample selection bias. The sampling consisted of 40 POS in the city of Barcelona. At least 2 public spaces of all 10 districts of the city were represented to try to avoid an important bias. However, as 20 of them were concentrated in Sants-Montjuïc, results needed to be interpreted carefully. Additionally, as with most studies using systematic observation there was the possibility of generating reactance on park users. To minimize this bias, observers had instructions of being in locations where low visibility to park users were guaranteed. Although very few people respond with curiosity, there was an episode where the observer was asked to stop recording and leave, reflecting appropriation processes of public space by certain communities that characterize some places.

This study is an example of the possibilities that systematic observation offers for the study of naturally occurred interactions in everyday life. We have also shown the informative potential of polar coordinate technique when analyzing big observational data with results in form of easy-to-understand maps. Our results have documented men and women preferences on park use, in unisex, and mixed groups. Together, these findings can help urban planners and policy-makers to assess and address specific gender needs associated with environmental justice. The approach can also provide relevant data to decide which parks need interventions or to examine the impact of park renovations on park use. Further research could also consider assessing social and environmental characteristics of POS and their implications on activity patterns and perceived insecurity.

## Ethics Statement

This study was carried out in accordance with the Declaration of Helsinki. Systematic observation was performed anonymously.

## Author Contributions

SV and FP-T developed the project and the design of the study. MTA supervised the methods. FP-T coordinated data collection and did the data analysis, closely supervised by MTA. FP-T did the writing of the article that was critically review by MTA and SV. All authors approved the final, submitted version of the manuscript.

### Conflict of Interest Statement

The authors declare that the research was conducted in the absence of any commercial or financial relationships that could be construed as a potential conflict of interest.

## Footnotes

^1^Source: Municipal Services Survey. Barcelona City Council.
